# Epidemiological and aetiological characteristics of hand, foot, and mouth disease in Sichuan Province, China, 2011–2017

**DOI:** 10.1038/s41598-020-63274-3

**Published:** 2020-04-09

**Authors:** Di Peng, Yue Ma, Yaqiong Liu, Qiang Lv, Fei Yin

**Affiliations:** 1 West China School of Public Health and West China Fourth Hospital, Sichuan University, Chengdu, Sichuan China; 2 Sichuan Center for Disease Control and Prevention, Chengdu, Sichuan China

**Keywords:** Viral infection, Epidemiology

## Abstract

Hand, foot, and mouth disease (HFMD) remains a threat to the Asia-Pacific region. The epidemiological characteristics and pathogen spectrum of HFMD vary with space and time. These variations are crucial for HFMD interventions but poorly understood in Sichuan Province, China, particularly after the introduction of the EV-A71 vaccine. Using descriptive methods, regression analyses, spatial autocorrelation analysis, and space-time scan statistics, we analysed the epidemiological and aetiological characteristics of HFMD surveillance data in Sichuan Province between 2011 and 2017 to identify spatio-temporal variations. The dominant serotypes of HFMD have changed from enterovirus 71 and coxsackievirus A16 to other enteroviruses since 2013. The seasonal pattern of HFMD showed two peaks generally occurring from April to July and November to December; however, the seasonal pattern varied by prefecture and enterovirus serotype. From 2011 to 2017, spatio-temporal clusters were increasingly concentrated in Chengdu, with several small clusters in northeast Sichuan. The clusters observed in southern Sichuan from 2011 to 2015 disappeared in 2016–2017. These findings highlight the importance of pathogen surveillance and vaccination strategies for HFMD interventions; future prevention and control of HFMD should focus on Chengdu and its vicinity.

## Introduction

Hand, foot, and mouth disease (HFMD) is an acute infectious disease that is prevalent in the Asia-Pacific region. HFMD is caused by more than 20 different enteroviruses; enterovirus 71 (EV-A71) and coxsackievirus A16 (Cox A16) were considered the most common aetiological agents of HFMD in the past^1,2^. Due to the high rate of gene mutation and recombination, the pathogen spectrum of HFMD is gradually changing, and antibodies against one enterovirus have no cross-protection against other enteroviruses^3^. Since 2008, other enteroviruses, such as Cox A6 and Cox A10, have been replacing EV-A71 and Cox A16 as the dominant serotypes, and large outbreaks caused by other enteroviruses have been reported in Europe and Asia^4–8^. In addition, dominant serotypes of HFMD also differ by regions; for example, Cox A16 and Cox A6 are dominant in India, EV-A71 and Cox A16 are dominant in Vietnam and EV-A71 is dominant in Singapore^9–11^. It is worth noting that the EV-A71 vaccine was licensed in China in 2016, and other vaccines are still under development^12^. The use of the EV-A71 vaccine possibly alters the pathogen spectrum of HFMD temporally^13^. Characterizing the serotypes temporally and spatially to understand the evolutionary dynamics of HFMD enteroviruses is crucial for vaccination strategies^3^.

HFMD is transmitted through contact with, inhalation of or ingestion of enterovirus-contaminated objects^14^. Transmission is influenced by environmental factors, particularly climatic factors, and socio-economic factors, which may lead to temporal and spatial heterogeneity in HFMD incidence^15,16^. Previous studies characterized the temporal features of HFMD according to seasonal patterns and revealed that the annual peaks differ among regions. In northern Asia, only one peak of HFMD, which frequently occurs from May to July, is usually observed per year, whereas two peaks occurring in late spring and winter are observed in southern Asia^1,17–20^. The spatial distributions of HFMD differ between provinces in mainland China and show spatial clustering^1^. Recognizing high-risk regions and time periods and understanding the spatio-temporal variations in HFMD would facilitate the development of better control policies for HFMD.

Sichuan Province is located in Southwest China; it has complex terrain and climate systems, leading to distinct epidemiological characteristics of HFMD compared to other regions^21,22^. A previous study briefly described the epidemiological characteristics and detected spatio-temporal clusters of HFMD in Sichuan Province from 2008 to 2013^23^. Considering the increase in the number of HFMD in Sichuan Province in recent years, the amount of data analysed in the previous study was relatively small, and the epidemiological characteristics of HFMD may have changed. More importantly, the study did not mention the aetiological characteristics of HFMD, and the pathogen spectrum of HFMD in Sichuan Province was not clear; this spectrum may be gradually changing, particularly because the EV-A71 vaccine became available in Sichuan Province in 2016.

Based on the HFMD surveillance data for Sichuan in 2011–2017, we provide comprehensive insight into the epidemiological and aetiological characteristics of HFMD using descriptive methods, regression analysis, spatial autocorrelation analysis and space-time scan statistics to identify high-risk areas and periods and dominant serotypes of HFMD to provide a basis for HFMD interventions.

## Results

### Demographic characteristics

The total incidence of HFMD was 79.67/100000 in Sichuan Province during 2011–2017. The morbidity of HFMD varied by age, sex and population classification. The age of the patients ranged from 0 to 94 years, with a median age of 2.24 (interquartile range: 1.45–3.27) years. HFMD patients aged under 5 years predominated, accounting for 96.64% of the 449485 cases (Table 1). Approximately 73.91% of HFMD patients were aged 1–3 years. The highest incidence of HFMD was observed in patients aged 1 year (2186.24/100000) and 2 years (1828.04/100000); the incidence of HFMD in adults (age ≥ 15 years) was fairly low (0.38/100000).

**Table 1:** Incidence rates (/100000) of HFMD in Sichuan Province, 2011–2017.

	2011	2012	2013	2014	2015	2016	2017	Mean
Total cases	38350	49000	51556	101031	63634	87585	58329	449485
Annual incidence	47.66	60.68	64.08	126.72	79.20	108.24	71.58	79.67
**Age (incidence)**								
< 1 year	4344 (467.29)	5689 (756.65)	7386 (794.52)	13713 (1781.54)	10072 (1298.62)	9490 (1214.99)	6781 (862.10)	57475 (1004.09)
1 year	10633 (1205.24)	14879 (1909.25)	16844 (1909.25)	32525 (3447.80)	20799 (2188.01)	28078 (2932.66)	18387 (1907.29)	142145 (2186.14)
2 years	10818 (1267.20)	13386 (1476.76)	12607 (1476.76)	25074 (2918.93)	15311 (1768.86)	20377 (2337.59)	13157 (1498.81)	110730 (1828.04)
3 years	7190 (859.02)	8589 (1028.20)	8606 (1028.20)	17021 (2141.08)	10321 (1288.43)	16676 (2067.17)	10927 (1345.96)	79330 (1384.78)
4 years	2929 (348.28)	3367 (392.03)	3297 (392.03)	6934 (900.19)	3750 (483.15)	7081 (905.91)	4911 (623.92)	32269 (573.69)
5 years	1091 (126.34)	1338 (140.93)	1217 (140.93)	2540 (362.69)	1526 (216.26)	2726 (383.60)	2012 (281.16)	12450 (233.32)
6–14 years	1207 (14.28)	1607 (16.65)	1408 (16.65)	2842 (34.98)	1575 (19.24)	2731 (33.12)	1902 (22.91)	13272 (22.80)
≥ 15 years	138 (0.21)	145 (0.29)	191 (0.29)	382 (0.57)	280 (0.42)	426 (0.63)	252 (0.37)	1814 (0.39)
**Sex (incidence)**								
Male	23602 (57.78)	30328 (74.00)	30523 (74.42)	59585 (147.97)	37733 (92.98)	51525 (126.08)	33890 (82.35)	267186 (93.57)
Female	14748 (37.23)	18672 (46.95)	21033 (53.10)	41446 (105.03)	25901 (65.13)	36060 (90.04)	24439 (60.60)	182299 (65.43)
Male-to-female ratio	1.60	1.62	1.45	1.44	1.46	1.43	1.39	1.47
**Population classification (%)**								
Scattered children	20819 (54.29)	31208 (3.69)	34802 (67.50)	66744 (66.06)	43908 (69.00)	57882 (66.09)	38000 (65.15)	293363 (65.27)
Nursery children	16271 (42.43)	16193 (33.05)	15467 (30.00)	31591 (31.27)	18209 (28.62)	27366 (31.25)	18740 (32.13)	143837 (32.00)
School-children	1032 (2.69)	1283 (2.62)	1124 (2.18)	2381 (2.36)	1278 (2.01)	1968 (2.25)	1370 (2.35)	10436 (2.32)
Others	228 (0.59)	316 (0.64)	163 (0.32)	315 (0.31)	239 (0.38)	369 (0.42)	219 (0.38)	1849 (0.41)

The male-to-female ratio of HFMD cases was 1.47:1 (ranging from 1.39:1 to 1.62:1) and showed a declining trend. The first and second highest proportion of classified HFMD cases were observed in scattered children and nursery children, respectively; the proportions of school-children and others were relatively small (Table 1).

### Enterovirus serotype distribution

**Figure 1:**
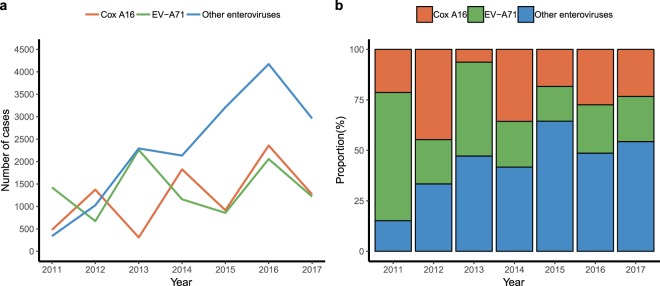
Enterovirus serotypes of laboratory-confirmed HFMD cases in Sichuan Province, 2011–2017. (**a**) The number of laboratory-confirmed cases. (**b**) The proportion of laboratory-confirmed cases.

Aetiological data showed 34313 (7.63% of total cases) laboratory-confirmed cases of HFMD in Sichuan Province during 2011–2017; 28.12%, 24.86% and 47.02% were EV-A71 positive, Cox A16 positive and other enteroviruses positive, respectively. The annual numbers and proportions of serotypes among laboratory-confirmed cases were not consistent from 2011 to 2017. The numbers of EV-A71 and Cox A16 cases fluctuated during the seven years, while the number of cases of other enteroviruses first trended upward and then decreased in the last year (Fig. 1a). EV-A71 dominated in 2011 (63.47%), and Cox A16 (44.73%) dominated in 2012. In 2013, other enteroviruses (47.16%) were present along with EV-A71 (46.48%) and accounted for most of the laboratory-confirmed cases (Fig. 1b). After 2013, EV-A71 and Cox A16 were replaced by other enteroviruses and were no longer dominant pathogens.

**Figure 2:**
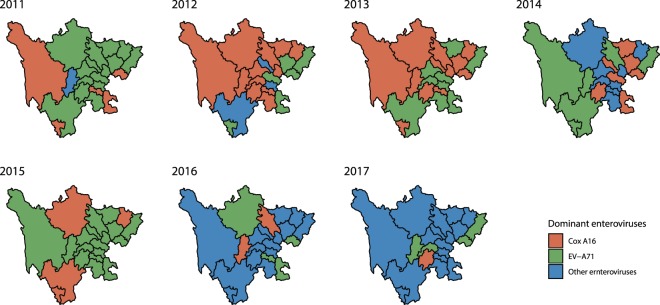
Dominant HFMD serotypes at the prefecture level in Sichuan Province, 2011–2017.

Figure 2 demonstrates that the prevalence of the dominant serotypes of HFMD at the prefecture level changed each year, and these dominant serotypes were replaced every 1–3 years in most areas.

### Temporal characteristics

**Figure 3:**
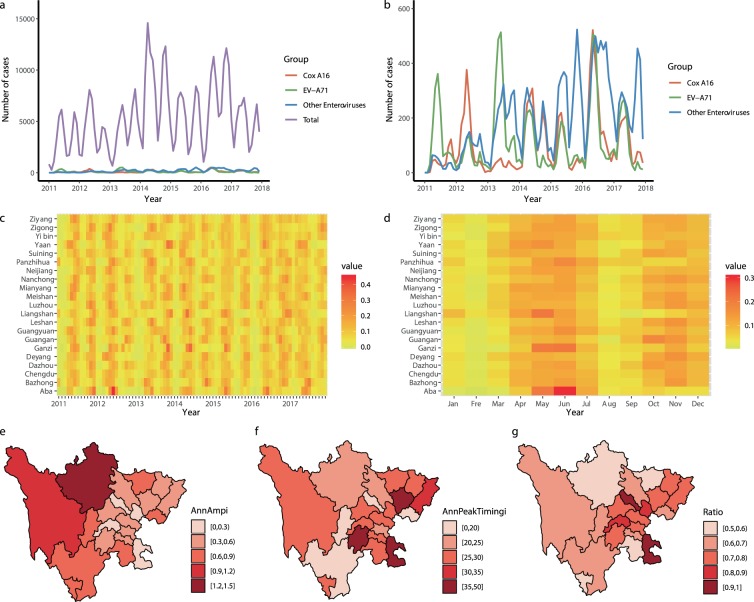
Temporal characteristics of HFMD in Sichuan Province, 2011–2017. (**a**) Time series of monthly cases of HFMD. (**b**) Time series of monthly laboratory-confirmed cases of HFMD. (**c**) Heatmap of monthly cases of HFMD at the prefecture level (standardized by the annual number of cases). (**d**) Heatmap of seasonal cases of HFMD at the prefecture level. (**e**) Annual amplitude of HFMD at the prefecture level based on seasonal multiple linear regression. (**f**) Annual peak times of HFMD at the prefecture level based on seasonal multiple linear regression. (**g**) Importance of semi-annual periodicities (ratio = semi-annual amplitude/(semi-annual amplitude + annual amplitude)).

The annual incidence of HFMD in Sichuan Province initially increased and then fluctuated from 2011 to 2017 (Table 1). HFMD presented a seasonal distribution with semi-annual peaks (Fig. 3). Two peaks were observed, one in April to July and the other in October to December, and the peaks accounted for 44.48% and 32.81% of all cases, respectively.

At the prefecture level, heatmaps of HFMD showed that the incidence of HFMD was bimodal (Figure c-d), and the results of the seasonal linear regression model also strongly suggested semi-annual periodicity (Figure g). HFMD had greater seasonal patterns in western and southern Sichuan Province than in other parts of the province, and the prefecture-level HFMD incidence did not peak simultaneously (Figure c-f). The spring peak in Ganzi, Aba and Panzhihua occurred one month later, and the winter peak time in Panzhihua and Liangshan occurred two months later than that in the other prefectures.

The results of the descriptive analyses and models showed that different serotypes peaked simultaneously but differed in their peak patterns. EV-A71 and Cox A16 showed a major and a minor peak in a year, while other enteroviruses with different serotypes presented double-peak distributions (see Supplementary Fig. S1).

### Spatial characteristics

**Figure 4:**
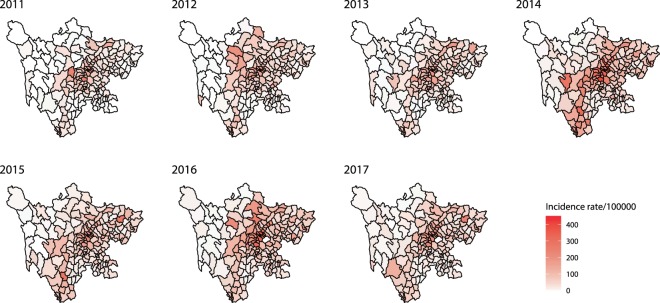
The incidence of HFMD at the county level in Sichuan Province, 2011–2017.

During 2011–2017, all counties in Sichuan Province reported the occurrence of HFMD. The spatial distribution of HFMD incidence was heterogeneous at the county level, ranging from 2.54/100000 (Shiqu) to 254.16/100000 (Qingbaijiang). The counties with the highest incidence of HFMD were Dongpo (2011), Jinniu (2012), Longquanyi (2013), Qingbaijiang (2014), Xichang (2015), Qingbaijiang (2016) and Luojiang (2017). Overall, the relatively high-incidence areas were Chengdu-centric and located in the northwest-southeast belt of Sichuan (Fig. 4).

The global spatial autocorrelation analysis of HFMD by year showed that the annual Moran’s *I* values ranged from 0.479 to 0.591 and were all statistically significant, indicating that global spatial autocorrelation of HFMD existed in Sichuan Province in 2011–2017 (see Supplementary Table S1).

### Spatio-temporal clusters

**Figure 5:**
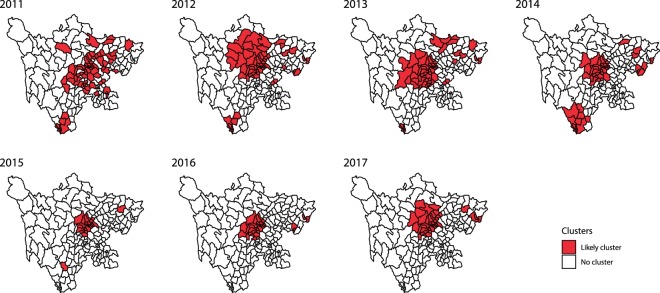
Spatio-temporal clusters of HFMD in Sichuan Province, 2011–2017 (considering the optimal maximum spatial cluster size).

Detailed information on the optimal value for the maximum spatial cluster size and spatio-temporal clusters in each year are provided in the supplemental data (see Supplementary Tables S2 and S3). The results of the scan statistics corresponding to the optimal selection of maximal window size suggested that the locations and sizes of clusters were partly similar and partly varied from year to year. Every year, the timing of the spatio-temporal clusters was concentrated at the peak of the HFMD incidence. The clusters were mainly located in the central part of Sichuan Province and increasingly concentrated in Chengdu (Fig. 5). From 2011 to 2015, some small clusters were identified in northeast and southern Sichuan; in 2016 to 2017, several small clusters existed in northeast Sichuan, but no cluster existed in southern Sichuan.

## Discussion

HFMD has produced epidemics in the Asia-Pacific region over the past two decades, and the incidence levels in China have remained high since the outbreak in 2008^24^. Our study presented the epidemiological and aetiological characteristics of HFMD more comprehensively than previous studies of HFMD in Sichuan Province. We found that HFMD mainly occurred in children under 5 years old, the dominant serotypes of HFMD were other enterovirus, HFMD peaked from April to May and from November to December, and the high-risk regions were concentrated in Chengdu.

Numerous studies have shown that more than 90% of HFMD cases occur in children, and affected males outnumber affected females^1,20,25^. However, the age of the susceptible population may be dynamic according to other studies of HFMD^26,27^. To understand these dynamics, HFMD surveillance must be continued, HFMD-susceptible populations should be analysed for an extended period, and vaccination should be recommended for susceptible children^28^. In addition, our study found that the male-to-female ratio of HFMD cases in Sichuan Province seemed to decline from 2011 to 2017; this result was not found in other studies, indicating that the incidence gap between males and females has narrowed. The decline was possibly due to more attention allocated by caregivers to the hygiene of boys than to the hygiene of girls, suggesting that health education was successful in Sichuan Province and should continue to be carried out in the future in Sichuan and other regions; this result deserves further investigation.

Few studies have investigated the pathogens responsible for HFMD in Sichuan Province, but understanding the enterovirus serotype alternation of HFMD is conducive to immunization implementation. Our study found that the dominant serotypes of HFMD converted from EV-A71 in 2011 and Cox A16 in 2012 to other enteroviruses in 2013 and beyond according to the surveillance data of Sichuan Province. The replacement of serotypes may be due to population immunity because antibodies induced by specific serotypes can provide protective immunity against only those specific serotypes^3,29,30^. After the introduction of the EV-A71 vaccine, however, the decline in HFMD caused by EV-A71 was not greater than that caused by Cox A16 in 2017, possibly because the EV-A71 vaccine was not widely implemented in the early stage of vaccination. Additional monitoring and molecular analyses of other enteroviruses in addition to EV-A71 and Cox A16 should be carried out to understand the changes in the pathogen spectrum and to develop vaccine strategies.

Our study revealed that the seasonal pattern of HFMD in Sichuan Province in southern China consisted of semi-annual peaks. At the prefecture level, temporal features differed within Sichuan and mainly manifested as inconsistent peak times, which may be due to distinct terrain and climatic factors. A previous study examining the relationship between HFMD and climatic factors in Sichuan Province illustrated that the incidence of HFMD was positively correlated with temperature (odds ratio = 2.59), relative humidity (odds ratio = 1.35) and sunshine hours (odds ratio = 1.06)^31^. During the four seasons, temperature and sunshine hours are obviously different in most prefectures in Sichuan Province; however, in the four prefectures (Ganzi, Aba, Liangshan, and Panzhihua), only humidity is significantly different, which is probably responsible for the delayed peak time. In addition, the seasonal patterns of EV-A71 and Cox A16 differed from those of other enteroviruses because the survival of enteroviruses is affected by environmental factors such as temperature, pH and sunlight^32,33^. Different enterovirus serotypes have varying tolerances to the environment, which may lead to different peak patterns of enteroviruses. At present, the influencing mechanisms of the associated factors are not clear and need further study.

The areas with high HFMD incidence were located from the northeast to southwest, with 1–3 clusters each year in Chengdu and northeast, southern or western Sichuan. The HFMD clusters varied among the 7 years in Sichuan Province according to parameters selected using MCS-P, mainly manifesting as a concentration of primary clusters and a disappearance of clusters in southern Sichuan. Studies have shown that the occurrence of HFMD is positively correlated with population density, population susceptibility and population mobility and negatively correlated with economic level and medical care^16,34^. Certain spatio-temporal clusters were scattered in northeast and southern Sichuan, possibly due to a low gross domestic product (GDP) and poor medical care^16^. Similar to other provinces whose HFMD clusters were located in their capitals, from 2011 to 2017, the main locations of clusters in Sichuan Province were increasingly concentrated in Chengdu; this concentration increase may be associated with the high population density and population mobility in Chengdu^35,36^. Chengdu and its vicinity are still key areas for the prevention and control of HFMD in Sichuan Province in the future.

Some limitations should be considered. First, not all HFMD cases in Sichuan Province were reported to the China Information System for Disease Control and Prevention; therefore, the incidence of HFMD may be underestimated and biased, although the monitoring of large-scale population data can still provide effective information for the prevention and control of HFMD^1,26^. Second, only 7.63% of reported HFMD cases in Sichuan Province from 2011 to 2017 underwent laboratory testing for pathogen identification for the purpose of determining the dominant circulating enteroviruses rather than verifying each HFMD case^37^. Third, we did not determine the enterovirus serotypes beyond EV-A71 and Cox A16 because that other enteroviruses had not been dominant in the past based on other studies.

In summary, HFMD has been and will be an important public health problem in Sichuan Province. The dominant serotype of HFMD in Sichuan Province has undergone dynamic changes, and other enteroviruses have recently assumed a dominant position. Thus, additional detailed molecular epidemiological analyses should be conducted for the development of vaccination strategies. The peak times of HFMD varied by prefecture, implying that the optimal time for intervention differs in different prefectures. Spatio-temporal clusters of HFMD in Sichuan Province showed dynamic changes, with increasing concentrations in Chengdu and disappearing concentrations in southern Sichuan. Our study should be helpful not only for the prevention and control of HFMD but also for the subsequent application of the MCS-P.

## Methods

### Study area

Sichuan Province is located in Southwest China (26.40°N~33.68°N and 98.31°E~107.99°E), covers an area of approximately 486000 square kilometres, and has a complex terrain consisting of mountains, hills, plains, basins and plateaus. More than 80 million people live in 21 prefectures and 180 counties in Sichuan Province. With a terrain that is low in the east and high in the west, significant regional climatic differences exist; the Sichuan basin has a subtropical humid climate, the mountainous southwestern region has a subtropical subhumid climate, and the northwest plateau region has an alpine climate.

### Case definitions and specimen testing

HFMD was listed as a Class C infectious disease in May 2008. Once an HFMD case is identified, medical institutions must report the case to the local Disease Control and Prevention Center (CDC) within 24 hours. HFMD cases were diagnosed according to the Chinese guidelines for the diagnosis and treatment of HFMD (2010 edition), and a clinical case was defined as a patient with papular or vesicular rash on hands, feet, mouth, or buttocks, with or without fever^1^. A laboratory-confirmed case was defined as a probable case with positive laboratory detection of specific nucleic acids, isolation, or a more than 4-fold increase in neutralizing antibodies (EV-A71, Cox A16 and other enteroviruses). Medical personnel or CDC personnel collected and sent samples (blood, throat swab, rectal swab, faeces, vesicular fluid, or cerebrospinal fluid) from clinical cases to the prefecture or provincial CDC for nucleic acid testing by real-time reverse transcription PCR (real-time RT-PCR). Laboratory test results were divided into 4 categories: EV-A71 positive, Cox A16 positive, other enteroviruses positive and enterovirus negative.

### Data collection

From January 1, 2011, to December 31, 2017, the surveillance data, including sex, birth date, population classification, address, date of symptom onset, type of diagnosis (clinical case or laboratory-confirmed cases) and laboratory test results, of 449485 HFMD cases in Sichuan Province were obtained from the China Information System for Disease Control and Prevention. The demographic information and specific ages of the residents of 180 counties were provided by the Sichuan Province Statistical Bureau to calculate incidence rates. Geographical boundary files for Sichuan Province were provided by the National Fundamental Geographic Information System of China.

### Analytical methods

Descriptive epidemiological methods were used to analyse the demographic (including age, sex and population classification), aetiological (EV-A71, Cox A16 and other enteroviruses), temporal and spatial characteristics of HFMD by year. Seasonal multiple linear regression models were fitted to weekly HFMD time series separately for each prefecture and each kind of enterovirus to quantify seasonality, including the amplitude (the maximum value minus the minimum value of a seasonal curve) and peak timing (see Supplementary Method 1)^1^. Global Moran’s *I* was calculated to quantify the spatial autocorrelation of HFMD in Sichuan Province between 2011 and 2017.

In addition, the widely used retrospective space-time scan statistic was utilized to detect annual spatio-temporal clusters of HFMD at the county level^38–41^. Spatio-temporal clusters of HFMD were defined as regions in which the incidence of HFMD abnormally increased during a certain period. The space-time scan statistic establishes a moving cylinder window with a dynamically changing radius and height corresponding to space and time, respectively. HFMD cases were randomly distributed under the null hypothesis^38^. The relative risk (RR) and log likelihood ratio (LLR) were calculated based on the incidence and populations inside and outside the window^38^. The space-time scan statistic can be conveniently applied with SaTScan software; however, the results of the space-time scan statistic are sensitive to the maximum spatial cluster size, which may impact the focus of intervention and the allocation of limited resources^42^. Thus, the selection of maximum spatial cluster sizes was important. When applying the space-time scan statistic with SaTScan, the maximum spatial cluster size changes from 1% to 50%. We utilized an index, maximum clustering set-proportion (MCS-P), to determine the optimal maximum spatial cluster size. MCS-P, which not only evaluates the most likely clusters, is an evaluation index proposed to evaluate the overall spatial accuracy of all detected statistically significant clusters^43^. Statistically significant clusters were included to construct a set for each result under the maximum spatial cluster size set, and all counties with an RR > 1 were added to construct a maximum clustering set (MCS)^43^. Then, the MCS-P was calculated as follows:

1 $${Z}_{i0}={\cup }_{j}{Z}_{ij},$$

2 $${Z}_{MCS}=\cup \{z|{p}_{z} > {q}_{z}\},$$

3 $$MCS-P=\frac{LLR({Z}_{i0})}{LLR({Z}_{MCS})},$$

where *Z*_*ij*_ is the *j*th statistically significant cluster; *Z*_*i*0_ is the whole of statistically significant clusters when the maximum spatial cluster size is *i*; *p*_*z*_ is the incidence inside the window; and *q*_*z*_ is the incidence outside the window. The MCS-P ranges from 0 to 1, and a larger MCS-P corresponds to increased spatial accuracy. Based on the MCS-P, the optimal maximum spatial cluster size was selected to obtain the final clusters.

Except for the space-time scan statistic, all other analyses were performed with R3.2.3 (https://www.r-project.org, package: “raster”, “spdep”). The space-time scan statistic was calculated with SaTScan 9.6 (http://www.satscan.org). All results were visualized with R3.2.3 (https://www.r-project.org, package: “ggplot2”, “rgdal”, “ggmap”). *P* values < 0.05 were considered statistically significant.

### Ethics statement

All patient information of HFMD surveillance data were collected from the China Information System for Disease Control and Prevention and this study was approved by the Ethics Committee of Sichuan Center for Disease Control and Prevention. All methods were carried out in accordance with relevant guidelines and regulations. No confidential information was included because analyses were performed at the aggregate level. All data employed in this study were deidentified prior to analysis and anonymized confidentiality, thus informed consent was not required.

## Supplementary information

Supplementary information.

## Data Availability

The datasets generated during and/or analysed during the current study are available from the corresponding author on reasonable request.
