# Supplementary material for: Epidemiological and aetiological characteristics of hand, foot, and mouth disease in Sichuan Province, China, 2011–2017
Source: Sci Rep. 2020 Apr 9;10:6117. doi: 10.1038/s41598-020-63274-3 (PMC7145801; doi:10.1038/s41598-020-63274-3)
Supplement: Supplementary file 1 — Supplementary information. [file 41598_2020_63274_MOESM1_ESM.pdf]

Epidemiological and aetiological characteristics of hand, foot, and mouth disease in Sichuan Province, China, 2011-2017

Di Peng [1†], Yue Ma[1†], Yaqiong Liu[2], Qiang Lv[2] & Fei Yin[1*]

[1] West China School of Public Health and West China Fourth Hospital, Sichuan University, Chengdu, Sichuan, China. [2] Sichuan Center for Disease Control and Prevention, Chengdu, Sichuan, China.

†: These authors contributed equally to this work and should be considered co-first authors.

*Correspondence: Fei Yin, PhD, West China School of Public Health and West China Fourth Hospital, Sichuan University, No. 16 Section 3, Renminnan Road, Chengdu, Sichuan 610041, People's Republic of China. (Email: westsilverhx@163.com).

Supplementary Method 1. Additional information regarding the estimation of seasonal parameters of seasonal multiple linear regression.

We used following linear regression model to estimate the peak timing and amplitude of the annual and semi-annual periodicities of HFMD activity in each prefecture, including harmonic terms representing annual and semi-annual periodicities [1]. We extracted the amplitude and peak timing of the annual and semiannual cycles based on model coefficients [2]. The amplitude measures the difference between the maximum and minimum of a seasonal curve [3]. The model was:

$$\text{hfmd}_i(t) = a_i + b_i * \cos(2\pi * t / 52.17) + c_i * \sin(2\pi * t / 52.17) + d_i * \cos(4\pi * t / 52.17) + e_i * \sin(4\pi * t / 52.17) + \varepsilon_i(t)$$

where $\text{hfmd}_i(t)$ are the weekly standardized counts of HFMD cases (or EV-A71, or Cox A16, or other enterovirus) isolates in prefecture i, where standardization is obtained by dividing weekly values by the annual number of HFMD cases, t is a running index for week, and a,b, c, d and e are the intercept and seasonal terms to be estimated from the data.

On the basis of the estimated model coefficients representing harmonic terms, we extracted the amplitude of annual and semi-annual periodicities (AnnAmp_i=sqrt(b_i^2+

c_i^2) and SemiAnnAmp$_i$= sqrt(d_i^2+e_i^2)), and the annual peak timing (AnnPeakTiming$_i$ = -atan(c_i / b_i)). To evaluate the relative importance of annual and semi-annual HFMD periodicities by prefecture, we calculated the ratio between the amplitude of the semi-annual periodicity and the sum of the amplitudes of annual and semi semi-annual periodicities. A ratio close to 1 is indicative of dominant semisemi-annual periodicity while a ratio close to 0 indicates dominant annual periodicity. The ratio is estimated as Ratio$_i$= SemiAnnAmp$_i$ / (SemiAnnAmp$_i$ + AnnAmp$_i$). To control for different levels of HFMD activity across prefectures, we compare the relative amplitudes of annual and semi semi-annual periodicity, obtained by dividing AnnAmpi and SemiAnnAmpi by the mean of the hfmd$_i$(t) time series.

Reference

1. Xing, W. J. *et al.* Hand, foot, and mouth disease in China, 2008-12: an epidemiological study. *Lancet Infect Dis* **14**, 308-318 (2014).

2. Yu H, Alonso WJ, *et al*. Characterization of regional infl uenza seasonality patterns in china and implications for vaccination strategies: spatio-temporal modeling of surveillance data. *PLoS Med* 10, e1001552 (2013).

3. Naumova EN, Jagai JS, *et al.* Seasonality in six enterically transmitted diseases and ambient temperature. *Epidemiol Infect* 135: 281–92 (2007).

Supplementary Table S1. The results of the spatial autocorrelation of HFMD in Sichuan Province, 2011-2017.

Year	Moran's *I*	Expected Index	Variance	*Z*-score	*P*
2011	0.559	-0.006	0.002	12.775	<0.001
2012	0.566	-0.006	0.002	12.82	<0.001
2013	0.51	-0.006	0.002	11.586	<0.001
2014	0.565	-0.006	0.002	12.803	<0.001
2015	0.479	-0.006	0.002	10.923	<0.001

2016	0.591	-0.006	0.002	13.408	<0.001
2017	0.553	-0.006	0.002	12.565	<0.001

Supplementary Table S2. The results of the largest MCS-P and optimal maximum spatial cluster size.

Year	MCS-P	Optimal maximum spatial cluster size
2011	0.7172	2
2012	0.7333	27-32
2013	0.7364	27-28
2014	0.5301	34-35
2015	0.7300	41-50
2016	0.8131	44-50
2017	0.8445	45-50

Supplementary Table S3. The most likely clusters of hand, foot, and mouth disease in Sichuan Province, 2011-2017 (considering the optimal maximum spatial cluster size).

Year	Cluster time	Number of counties	Observed cases	Expected cases	RR	LLR	*P*
2011							
1	2011/4/16-2011/7/1	3	1148	127.59	9.24	1515.42	<0.0001
2	2011/10/15-2011/12/29	2	1003	150.71	6.81	1058.37	<0.0001
3	2011/6/15-2011/12/13	1	1323	285.38	4.77	1005.91	<0.0001
4	2011/6/16-2011/12/14	1	1256	257.58	5.01	1004.71	<0.0001
5	2011/4/12-2011/7/15	1	829	107.52	7.86	978.62	<0.0001
6	2011/10/14-2011/12/27	4	896	156.62	5.83	830.56	<0.0001
7	2011/10/14-2011/12/31	2	853	150.69	5.77	782.88	<0.0001
8	2011/3/30-2011/8/2	1	759	127.63	6.05	727.06	<0.0001
9	2011/4/8-2011/8/1	2	898	183.76	4.98	717.19	<0.0001
10	2011/3/24- -2011/7/10	2	669	138.48	4.90	526.89	<0.0001
11	2011/4/9-2011/6/21	8	662	139.53	4.81	511.86	<0.0001
12	2011/4/15-2011/8/5	2	835	219.04	3.87	506.41	<0.0001
13	2011/4/23-2011/7/17	1	689	167.58	4.17	456.25	<0.0001
14	2011/3/25-2011/6/25	2	422	83.18	5.12	348.02	<0.0001
15	2011/5/2-2011/7/14	1	449	105.94	4.28	306.90	<0.0001
16	2011/4/6-2011/7/11	1	567	163.44	3.51	303.89	<0.0001
17	2011/6/24-2011/12/22	2	803	297.34	2.74	295.47	<0.0001
18	2011/6/7-2011/12/5	2	672	279.41	2.43	199.17	<0.0001

19	2011/3/23-2011/6/14	2	384	127.61	3.03	167.52	<0.0001
20	2011/10/30-2011/12/26	1	123	19.74	6.25	121.92	<0.0001
21	2011/6/15-2011/7/13	1	148	42.26	3.51	79.92	<0.0001
22	2011/9/17-2011/12/1	1	88	21.06	4.19	58.96	<0.0001
23	2011/6/13-2011/11/29	1	120	37.95	3.17	56.18	<0.0001
24	2011/6/10-2011/11/19	1	226	101.93	2.22	56.08	<0.0001
25	2011/6/23-2011/7/14	1	101	29.94	3.38	51.82	<0.0001
26	2011/5/31-2011/7/4	1	107	33.26	3.22	51.35	<0.0001
27	2011/5/9-2011/7/26	2	187	81.59	2.30	49.84	<0.0001
28	2011/6/11-2011/7/6	1	100	31.39	3.19	47.32	<0.0001
29	2011/6/6-2011/7/22	2	109	39.96	2.73	40.39	<0.0001
30	2011/10/30-2011/12/7	1	38	6.29	6.04	36.63	<0.0001
31	2011/12/6-2011/12/31	3	85	29.32	2.90	34.82	<0.0001
32	2011/10/26-2011/11/28	1	129	57.54	2.25	32.76	<0.0001
33	2011/10/23-2011/12/6	1	110	46.70	2.36	30.99	<0.0001
34	2011/11/8-2011/12/16	1	101	42.80	2.36	28.57	<0.0001
35	2011/6/23-2011/7/17	1	70	27.30	2.57	23.23	<0.0001
36	2011/11/24-2011/12/12	1	42	13.27	3.17	19.67	0.006
37	2011/6/28-2011/7/1	1	20	3.59	5.57	17.94	0.0028
38	2011/9/25-2011/11/14	2	105	56.25	1.87	16.81	0.0075
2012							
1	2012/3/4-2012/7/24	46	17022	4994.23	4.69	10663.26	<0.0001
2	2012/3/25-2012/7/22	2	1020	277.18	3.74	591.81	<0.0001

3	2012/4/25-2012/7/21	6	617	209.33	2.97	260.97	<0.0001
4	2012/5/28-2012/7/18	2	271	59.56	4.57	199.63	<0.0001
5	2012/10/13-2012/12/13	1	283	74.88	3.80	168.58	<0.0001
6	2012/5/19-2012/7/28	2	327	107.09	3.07	145.61	<0.0001
7	2012/3/24-2012/7/1	1	449	187.35	2.41	131.50	<0.0001
8	2012/5/6-2012/7/10	1	158	76.61	2.07	33.05	<0.0001
9	2012/10/15- 2012/10/31	1	71	27.83	2.55	23.35	<0.0001
2013							
1	2013/6/12-2013/12/10	46	19169	6848.82	3.86	9288.26	<0.0001
2	2013/3/23-2013/6/17	1	733	104.98	7.07	800.31	<0.0001
3	2013/6/7- 2013/12/4	5	1682	584.75	2.94	691.77	<0.0001
4	2013/5/17-2013/10/20	1	512	147.42	3.50	274.19	<0.0001
5	2013/3/30-2013/7/17	2	648	241.60	2.70	234.54	<0.0001
6	2013/5/10-2013/7/18	3	343	96.73	3.56	188.48	<0.0001
7	2013/3/23-2013/5/3	1	200	53.66	3.74	117.00	<0.0001
8	2013/4/23-2013/6/16	2	173	83.76	2.07	36.32	<0.0001
9	2013/1/1-2013/1/12	1	34	9.08	3.75	19.98	0.0015
2014							
1	2014/3/9-2014/7/26	34	25651	9020.41	3.47	11779.79	<0.0001
2	2014/4/12-2014/7/13	10	3832	1009.08	3.91	2330.52	<0.0001
3	2014/10/20-2014/12/19	2	1070	366.95	2.94	444.51	<0.0001

4	2014/5/14-2014/7/20	1	498	122.92	4.07	322.36	<0.0001
5	2014/4/4-2014/7/1	1	415	134.26	3.10	187.99	<0.0001
6	2014/9/15-2014/11/9	4	815	484.41	1.69	93.96	<0.0001
2015							
1	2015/3/24-2015/7/12	25	9766	2902.74	3.79	5388.66	<0.0001
2	2015/3/24-2015/7/12	1	1583	173.16	9.35	2108.94	<0.0001
3	2015/1/3-2015/7/3	1	1257	319.03	4.00	792.62	<0.0001
2016							
1	2016/5/30-2016/11/28	31	27613	9303.22	3.87	14062.15	<0.0001
2	2016/9/15-2016/11/1	2	550	128.27	4.31	379.98	<0.0001
3	2016/9/26-2016/12/31	2	841	312.52	2.71	305.64	<0.0001
2017							
1	2017/3/12-2017/7/21	39	14932	5259.48	3.47	6849.15	<0.0001
2	2017/6/4-2017/8/10	1	1217	109.26	11.35	1836.34	<0.0001
3	2017/3/27-2017/7/9	3	1194	426.80	2.84	466.22	<0.0001

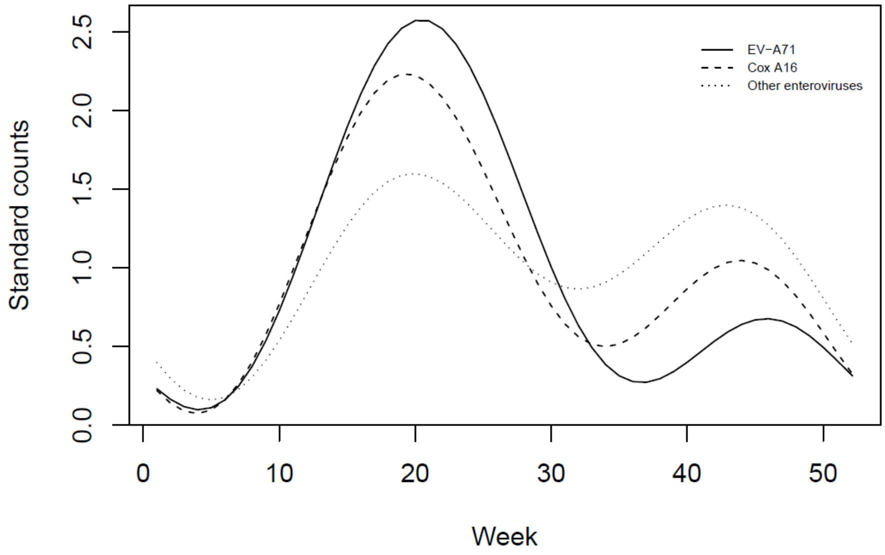

Supplementary Figure S1. The fitted seasonal curve of different serotypes of HFMD.
